# Vancomycin Therapeutic Drug Monitoring, Clinical Outcomes and Population Pharmacokinetic Model Evaluation in Neonates

**DOI:** 10.3390/children13050649

**Published:** 2026-05-06

**Authors:** Erin Chung, Najla Tabbara, Winnie Seto, Vibhuti Shah

**Affiliations:** 1 The Hospital for Sick Children, 555 University Avenue, Toronto, ON M5G 1X8, Canada; 2 Child Health Evaluative Sciences, SickKids Research Institute, 686 Bay Street, Toronto, ON M5G 0A4, Canada; 3 Leslie Dan Faculty of Pharmacy, University of Toronto, 144 College Street, Toronto, ON M5S 3M2, Canada; 4 Department of Pharmacy, Mount Sinai Hospital, 600 University Avenue, Toronto, ON M5G 1X5, Canada; 5 Institute of Health Policy, Management and Evaluation, University of Toronto, 155 College Street, Toronto, ON M5T 3M6, Canada; 6 Department of Paediatrics, Mount Sinai Hospital, 600 University Avenue, Rm 19-231N, Toronto, ON M5G 1X5, Canada

**Keywords:** vancomycin, neonates, neonatal intensive care unit, therapeutic drug monitoring, population pharmacokinetics, pharmacokinetic modeling

## Abstract

**Highlights:**

**What are the main findings?**

- Only 28% of neonates achieved target vancomycin trough concentrations (10–15 mg/L) with current hospital formulary dosing, indicating suboptimal precision in current therapeutic drug monitoring practices.
- A locally developed population pharmacokinetic (popPK) model outperformed 32 published models in predicting vancomycin exposure, demonstrating superior accuracy and reliability in a neonatal cohort.

**What are the implications of the main findings?**

- Higher vancomycin trough concentrations (>15 mg/L) did not improve infection outcomes but were associated with a significantly increased risk of acute kidney injury. Thus, we highlight considerations for a lower trough target near 10 mg/L, carefully balancing against individual risk factors, and close kidney function monitoring while on vancomycin therapy.
- Model-informed precision dosing using a validated popPK model offers a safer and more effective approach to optimize vancomycin therapy in neonates. This model should be considered for broader implementation in neonatal intensive care units.

**Abstract:**

**Background/Objectives:** Vancomycin dosing in neonates is challenging due to high pharmacokinetic variability and immature renal function. This study evaluated current therapeutic drug monitoring (TDM) practices, the association between vancomycin concentration and clinical outcomes, and the predictive performance of a locally developed population pharmacokinetic (popPK) model compared to the published models. **Methods:** This was a retrospective cohort study of neonates admitted to a tertiary neonatal intensive care unit (NICU). We assessed the persistent positive culture, infection recurrence, mortality and acute kidney injury (AKI) stratified by initial vancomycin trough concentrations (<10 mg/L, 10–15 mg/L, >15 mg/L). The locally developed popPK model was externally validated and compared with 32 other published neonatal vancomycin popPK models (with a total of 33 models evaluated). **Results:** A total of 366 neonates were included (mean postmenstrual age of 28.9 ± 3.81 weeks; 191 received at least 5 days of vancomycin). Only 28% of neonates achieved initial vancomycin trough concentrations within the 10–15 mg/L using standard vancomycin dosing. Higher vancomycin trough concentrations (>15 mg/L) were not associated with improved efficacy but were significantly associated with a higher incidence of AKI. The locally derived popPK model demonstrated superior predictive accuracy, meeting all predefined performance criteria, whereas none of the 32 other published models met all the criteria. **Conclusions:** Current vancomycin dosing strategies often result in suboptimal exposure and increased nephrotoxicity without added efficacy. Model-informed precision dosing using the locally developed popPK model may offer a safer, more effective approach for neonatal vancomycin therapy.

## 1. Introduction

Vancomycin is one of the most common antibiotics used in the treatment of neonatal infections, particularly those caused by coagulase-negative staphylococci (CoNS) or methicillin-resistant *Staphylococcus aureus* (MRSA) [1,2]. Despite its widespread use, vancomycin dosing in neonates remains highly challenging due to significant inter-individual pharmacokinetic (PK) variability, and the ethical constraints of conducting high-quality PK studies in this population [3,4,5,6]. Vancomycin therapeutic drug monitoring (TDM) has traditionally relied on trough concentration target ranges derived from adult MRSA pneumonia individuals with a trough range of 15–20 mg/L used for serious MRSA infections with higher minimum inhibitory concentrations (MICs) > 1 mg/L [7]. In contrast, vancomycin is most frequently used to treat CoNS sepsis in neonates, which typically exhibits lower MICs ≤ 0.5 mg/L [8] and can have different pharmacodynamic targets [9]. Consequently, achieving the higher adult trough targets in neonates may expose them to unnecessary nephrotoxicity without providing additional therapeutic benefits [10,11,12]. Furthermore, the rapid growth and physiological development (glomerular filtration maturation, body water composition shifts, and protein binding variations) during the neonatal period influences vancomycin pharmacokinetics and pharmacodynamics that are not observed in adults. This makes it critical to fill the knowledge gap in defining a specific vancomycin target range for neonates, rather than relying on adult data [12].

While previous studies have mentioned broad vancomycin trough ranges of 5–20 mg/L [6,13,14,15], these recommendations lacked a rigorous evaluation of their association with clinical effectiveness. Our prior work, utilizing multivariable regression and machine learning algorithms, have identified a more precise trough target range of 10–15 mg/L that is sufficient to achieve the necessary area-under-the-concentration-time curve/MIC (AUC/MIC) ratio, and optimizes efficacy (persistent or recurrent infections) while minimizing acute kidney injury (AKI) risk [8]. However, given that this finding was derived from a single-center dataset, further evaluation in an independent cohort is essential to confirm its generalizability.

Recent vancomycin dosing and monitoring guidelines have advocated for a shift toward model-informed precision dosing (MIPD) using population pharmacokinetic (popPK) models [10,16,17,18]. These models can inform individualized dosing recommendations to attain target exposures faster and more reliably with fewer dose adjustments. Recently, using rigorous and comprehensive covariate and popPK analysis, we developed an institution-specific neonatal vancomycin model based on 442 neonates [19]. This study identified body weight, postmenstrual age (PMA), and serum creatinine (SCr) as being the three most significant predictors of vancomycin pharmacokinetics. Based on this model, we derived initial vancomycin dosing regimens, using Monte Carlo simulations to achieve a target range of 10–15 mg/L [19]. This model was implemented via a dosing calculator (https://app.firstline.org/en/clients/41-sickkids/antimicrobials/14631-vancomycin/dosing/97-neonatal, accessed on 27 April 2026) and prospectively evaluated in a quality improvement project within the same institution. We demonstrated improved target attainment, reduced the need for dose adjustments and reduced painful and invasive monitoring of vancomycin concentrations via bloodwork [20]. However, because this popPK model was derived from a single institution, its application is inherently limited to the institution where it was developed. Another significant knowledge gap was the lack of externally validated models that demonstrate a robust predictive performance across diverse clinical settings. A primary limitation of applying institution-specific models to external populations is that a model developed in one setting may inadvertently capture site-specific factors, such as unique demographic or clinical characteristics, fluid management protocols, local patterns of concomitant medication use, or specific assay characteristics that do not translate to other NICUs. Consequently, relying on such models without external validation poses a significant risk of prediction error and suboptimal dosing for neonates.

While several studies have developed and externally validated neonatal vancomycin popPK models, their external predictive performance varied widely [19,21,22]. This inconsistency likely arises from demographic and clinical feature heterogeneity (e.g., differences in age and illness severity), and methodological variations (e.g., inconsistent evaluation metrics used, covariate selection strategies). Hence, even when external validation has been performed previously, the divergent conclusions across studies underscore the need to validate models within specific cohorts.

Beyond model performance, other significant barriers may hinder widespread adoption. These barriers include clinical workflow integration into daily practice, MIPD software availability and usability (access to user-friendly, bedside calculators), clinician training (building trust and proficiency in interpreting Bayesian forecasts), and laboratory test accessibility and turnaround times to reap the benefits of a real-time dosing schedule. Addressing these operational and educational barriers, alongside rigorous model validation, is essential for the implementation of precision dosing in neonatal care.

The primary objective of this study was to describe vancomycin dosing, serum vancomycin concentrations, and the incidence of clinical outcomes (persistent positive culture, infection recurrence, mortality, and AKI) in neonates. The secondary objectives were to investigate the association between initial vancomycin trough concentrations and these clinical outcomes; externally validate a locally developed popPK model in a cohort of neonates admitted to NICU; and compare its predictive performance with 32 other published popPK models.

## 2. Materials and Methods

**Study design and population:** We conducted a retrospective cohort study of neonates who received intravenous vancomycin between 1 October 2016, and 31 December 2021, and had at least one serum vancomycin concentration measured in a Level III Neonatal intensive care unit (NICU) at Mount Sinai Hospital (Toronto, Canada). Neonates with a PMA of ≥44 weeks and a postnatal age (PNA) of ≥28 days, or without vancomycin TDM, were excluded. This exclusion is a methodological necessity, as observed vancomycin concentrations are required to evaluate its association with outcomes, calculate prediction errors and validate the model performance. In our routine clinical practice, TDM is a standard of care for any neonate completing a full course of vancomycin; thus, the absence of TDM typically indicates a very short duration of therapy or early discontinuation, rather than a systematic omission of a specific neonate subgroup. Additionally, infants with a PMA > 44 weeks or PNA > 28 days were excluded, as vancomycin pharmacokinetics in this age range begin to diverge from the neonatal maturation trajectory, and clinical management often transitions to pediatric dosing protocols. This study was approved by both Mount Sinai Hospital and The Hospital for Sick Children (SickKids) Research Ethics Boards with a waiver of informed consent.

**Vancomycin dosing and monitoring practice:** Each neonate was prescribed vancomycin using weight- and PMA-based dosing guidelines with TDM. The hospital’s standard initial intravenous vancomycin dosing regimen comprised 10 mg/kg/dose, with the dosing interval determined by PMA (e.g., 10 mg/kg q12h for PMA < 30 weeks, increasing to q8h for PMA ≥ 30 weeks). These dosing regimens reflect our local protocol optimized for our neonatal population, which may vary from other dosing references. Vancomycin serum concentrations were measured using an immunoturbidimetric assay on the Roche cobas platform, with a lower limit of quantification of 4 mg/L. Vancomycin concentrations < 4 mg/L were replaced with 2 mg/L. The SCr levels were analyzed using the enzymatic creatinine method on the same platform. Vancomycin trough concentrations were typically obtained before the fourth dose, which is in keeping with standard neonatal TDM practice. While this approximates the steady state for many neonates, based on a median half-life of 7.3 h [19], pharmacokinetic variability means that a true steady state is not universal, but evaluating target attainment at this timepoint is critical to ensure therapeutic concentrations and optimize safety and efficacy, even during the non-steady-state phase. During the study period, the target trough ranges used in clinical practice were 10–15 mg/L for non-central nervous system infections. Dose adjustments were made at clinicians’ discretion, based on TDM.

**Data collection and outcomes:** Data were obtained from the electronic health record (EHR) system, including demographics (sex, gestational age, PNA, PMA, weight at start of vancomycin therapy), vancomycin dosing details, SCr, urine output, serum vancomycin concentration, indication for vancomycin use, and concomitant use of nephrotoxic drugs. Clinical outcomes were also collected for each vancomycin treatment course: (1) persistent positive culture, defined as repeated positive culture ≥ 7 days after vancomycin therapy initiation; (2) recurrence, defined as the isolation of the same organism in cultures taken after the completion of the first vancomycin course for a culture-proven infection within 30 days of the end of treatment; (3) 30-day all-cause mortality; and (4) AKI during vancomycin therapy, assessed by modified neonatal RIFLE criteria [23] (Supplementary Table S1). We defined AKI (stage 1 or above) as an increase in SCr by ≥50% from the baseline, SCr rise of ≥27 µmol/L, or oliguria <1 mL/kg/h. The baseline SCr was taken as the value prior to or at vancomycin initiation. We also noted the length of NICU stay and other relevant clinical parameters (inflammatory markers, concurrent nephrotoxic drug use) for descriptive analysis. If SCr was missing, AKI assessment relied on urine output criteria and serial SCr trends during the treatment course, ensuring comprehensive safety evaluation for the entire cohort.

**Statistical analysis:** Descriptive statistics were used to summarize neonatal characteristics, vancomycin dosing and serum concentrations. Continuous variables are reported as the mean (±standard deviation (SD)) or median (interquartile range (IQR)), based on data distribution and categorical variables as counted (%). The normality of continuous variables was assessed using a visual inspection of histograms. As most variables exhibited non-normal distributions, non-parametric tests were used. Neonatal characteristics and outcomes were compared for each analysis, using one of the following: Wilcoxon rank-sum for 2 groups, the Kruskal–Wallis rank sum test for >2 groups for continuous variables or Fisher’s exact test for categorical variables. To account for potential confounders related to illness severity, our effectiveness analysis was adjusted for PMA and the Score for Neonatal Acute Physiology II (SNAPPE-II), similar to our previous study [8]. A two-sided *p* < 0.05 was considered significant. These analyses were performed with R (R Core Team; R Foundation for Statistical Computing; v4.4.1; Vienna, Austria) statistical software.

**PopPK model identification:** A popPK model was previously developed and internally evaluated in a cohort of neonates admitted to SickKids Level IV NICU by some of the coinvestigators of the current study. The final popPK equations for the typical clearance (*CL_i_*) and volume of distribution (*V_i_*) are shown in the equations below: (1) and (2), respectively, where *WT* is weight in kg, *PMA* is postmenstrual age in weeks, and *SCr* is serum creatinine in µmol/L:

(1) $$CL_{i}=13.9\cdot (WT/70{)}^{1}\cdot \frac{PMA^{0.739}}{PMA^{0.739}+47.7^{0.739}}\cdot (SCr/34{)}^{-0.653}$$

(2) $$V_{i}=65.5\cdot (WT/70{)}^{1}$$

The details of the model development and internal evaluation are published [19]. This model is being externally validated, using the current neonatal cohort as the independent validation dataset.

A literature search was performed to identify other vancomycin popPK models in neonates published up to 31 December 2024. Sources included a prior published systematic review of neonatal and pediatric vancomycin popPK models (covering up to 17 August 2020) [24] and an updated Medline and Embase search using the same literature search approach as the published review (17 August 2020 up to 31 December 2024). We utilized the inclusion/exclusion criteria established in our previous work with a focus on the neonatal population [24]. For each included study, the structural model assumptions (one- vs. two-compartment) and covariate equations for clearance (CL) and volume of distribution (V) were extracted.

**External predictive performance evaluation:** Using the baseline demographic, clinical characteristics, vancomycin dosing and concentrations collected from each individual neonate, the model predictive performance was evaluated using population predictions (a priori) only. The dosing history for each neonate was reconstructed using the EHR data, specifically the recorded start and stop times, and dosing intervals of each vancomycin regimen. Consistent with the institutional protocol, all doses were assumed to be administered as a 1 h intravenous infusion. The sampling times were extracted directly from the patient charts. The model-predicted concentrations (*C*_pred_) were calculated for each observed sampling time point, using the neonate’s specific covariates (e.g., weight, PMA, SCr) and the reconstructed dosing history, following the Sawchuk–Zaske equation for short infusion kinetics [25].

The prediction error (PE) for each vancomycin concentration was defined as PE = *C*_pred_ − *C*_obs_, and the mean prediction error (ME) across all observations reflects the bias. Precision was assessed by the root mean squared error (RMSE), relative mean prediction error (Rel ME) and relative median prediction error (Rel MdE). The proportion of predictions within 30% of the observed value (p30%) was also used as an indicator of clinical accuracy. For each model, 95% confidence intervals (CI) for each metric were obtained by a nonparametric bootstrap (1000 resamples). Visual residual diagnostics of the top performing popPK models were also used, including *C*_obs_ vs. *C*_pred_ (or DV vs. PRED), residual or conditional weighted residual vs. time, and residual or conditional weighted residual vs. *C*_pred_ (or residual vs. PRED) with whole population and within a 7-day course of vancomycin. A visual predictive check (VPC) with 1000 simulations was used to assess whether the observed concentrations fell within the simulation percentiles. Simulations for the VPC utilized the actual dosing records and sampling times of the study cohort to account for the variability in dosing and sampling schedules.

Predefined criteria for acceptable performance were based on prior popPK model external evaluation [21,22] and consensus from study investigators: ME within ±0.5 mg/L, Rel ME ± 15%, Rel MdE ± 15%, RMSE < 10, and p30% > 45%. A ME within ±0.5 mg/L was selected because in the context of a therapeutic target range of 10–15 mg/L, an average deviation of 0.5 mg/L represents a clinically acceptable variability that is unlikely to result in inappropriate dosing decisions. The RMSE threshold of <10 was chosen to reflect acceptable precision across the wide dynamic range of observed concentrations in this cohort (spanning from <4 to 68.7 mg/L). The Rel ME, Rel MdE, and p30% thresholds were similar and were adapted from recent neonatal vancomycin model validation studies [21,22], representing a reasonable expectation for clinical accuracy in a heterogeneous population. Models meeting all five criteria were considered to be highly predictive, and those meeting at least three criteria were considered to be moderately predictive. The number of criteria met by each model was recorded. We identified the top-performing models and compared their performance metrics. Additionally, we performed subgroup analyses of the prediction error in strata of PNA (≤7 days vs. >7 days), PMA based on five developmental groups (<28 weeks = extremely preterm; 28 to <32 weeks = very preterm, 32 to <34 weeks = moderately preterm, 34 to <37 weeks = late preterm, and ≥37 weeks = term), weight (<1.5 kg vs. ≥1.5 kg), and SCr (<90 µmol/L vs. ≥90 µmol/L) to evaluate the top performing model generalizability across the neonatal sub-populations. Group comparisons were performed using the Mann–Whitney U test for binary strata and the Kruskal–Wallis test for multi-group PMA strata. All model simulations and error calculations were executed using R (v4.3.3) and Phoenix WinNonLin/NLME (Version 8.7, Certara, Radnor, PA, USA).

## 3. Results

### 3.1. Neonatal Characteristics

A total of 521 neonates were prescribed vancomycin during the study period. After applying the exclusion criteria (one infant with PMA > 44 weeks and a PNA > 28 days and 154 neonates without vancomycin TDM), 366 neonates with 661 vancomycin concentrations (median 1 [1,2] per neonate) were included. Table 1 presents the demographic and clinical characteristics of the study cohort compared to the previous cohort [19], which was used for model development. The current cohort of neonates had significantly lower bodyweight and GA (26.4 ± 3.2 weeks vs. 29.9 ± 5.5 weeks, *p* < 0.001), with a higher proportion of extremely preterm infants. Furthermore, the neonates had higher baseline SCr (median 56 µmol/L vs. 29 µmol/L, *p* < 0.001) and more frequent exposure to nephrotoxic co-medications (e.g., aminoglycosides in 63% vs. 52%, *p* = 0.003) compared to the previous cohort. There were fewer confirmed intra-abdominal infections (e.g., necrotizing enterocolitis or peritonitis) (26%), but more patent ductus arteriosus diagnoses (43%).

Indications for vancomycin in this cohort were predominantly suspected late-onset sepsis. Among the 521 neonates prescribed vancomycin, 191 neonates with 216 vancomycin courses had ≥5 days of therapy. Coagulase-negative Staphylococcus was the most common isolated organism from blood (79/216; 36.6%), urine (17/158; 10.7%), and cerebrospinal fluid (7/130; 5.4%) cultures.

### 3.2. Initial Vancomycin Troughs and Target Attainment

Using standard institutional dosing, the distribution of initial vancomycin trough concentrations was wide, ranging from <4 mg/L (limit of quantification of laboratory assay) to 68.7 mg/L. The overall median initial vancomycin trough concentration was 8.9 mg/L (IQR 6.3–12.2). Among the vancomycin courses with a duration of ≥5 days (*n* = 216), only 28% (*n* = 61) achieved a vancomycin trough concentration within the 10–15 mg/L range in the first dosing regimen. The majority (61%, *n* = 131) had initial vancomycin trough concentrations below 10 mg/L, while about 11% (*n* = 24) exceeded 15 mg/L. Among the 191 neonates, the initial vancomycin concentrations varied with maturity. Neonates with younger GA were noted to have a vancomycin trough >15 mg/L and neonates with higher GA were noted to have vancomycin trough <10 mg/L (*p* = 0.008). The concentrations also varied with severity of illness at birth (lowest SNAPPE II in the <10 mg/L group, *p* < 0.001) (Table 2). Furthermore, higher percentage of initial vancomycin trough concentrations >15 mg/L was observed in neonates with higher baseline SCr, lower urine output, and concurrent use of furosemide (Table 3). Lower initial vancomycin trough concentrations < 10 mg/L were observed in neonates with greater PMA and weight, lower baseline SCr and higher urine output. After TDM, among the vancomycin courses with a duration of ≥5 days (*n* = 216), 62% (*n* = 134) achieved a vancomycin trough concentration within the 10–15 mg/L range after dosage individualization.

### 3.3. Clinical Outcomes Versus Vancomycin Trough Concentrations

Of the 216 treatment courses, 3.7% (*n* = 8) had persistent positive culture, 0.46% (*n* = 1) had recurrent infection, and the 30-day mortality was 6% (*n* = 13). One neonate had concurrent persistent positive culture and recurrent infection, while another had persistent positive culture that coincided with death. We found no statistically significant association between the initial vancomycin trough and effectiveness outcomes (Table 4). Persistent positive cultures occurred in 4.6% of neonates with the initial trough <10 mg/L, 3.3% with trough 10–15 mg/L, and 0% with trough >15 mg/L (*p* = 0.88). After adjusting for PMA and SNAPPE-II, higher initial trough concentrations did not predict better odds of a cure. Compared to the initial vancomycin trough <10 mg/L, vancomycin trough concentrations within 10–15 mg/L were not significantly associated with reduced persistent positive cultures or mortality (adjusted odds ratio (OR) 0.48 [95% CI 0.08, 2.74] and 1.85 [95% CI 0.50, 6.83], respectively). Similarly, no difference in the risk of all-cause mortality (OR 1.49 [95% CI 0.25, 8.75]) was observed in >15 mg/L versus <10 mg/L.

During therapy, 10 neonates (4.6%) developed AKI. Higher vancomycin trough concentrations were significantly associated with an increased incidence of AKI (*p* < 0.001). AKI was highest in neonates with vancomycin trough concentrations > 15 mg/L (20.8%), compared to <10 mg/L (0.8%) and 10–15 mg/L (6.6%). Most AKI cases were mild to moderate (Stage 1–2).

### 3.4. Predictive Performance of Population Pharmacokinetic Models

**Figure 1:**
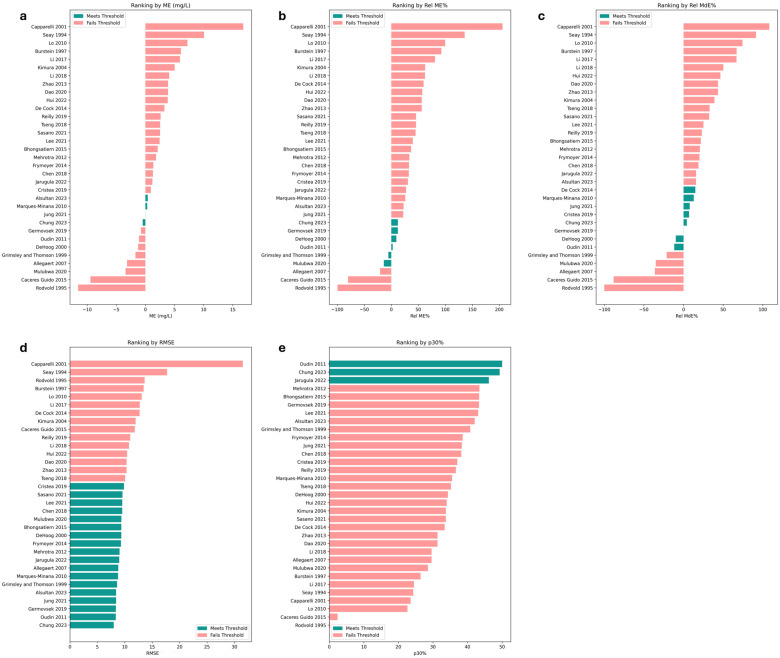
Plots of the predictive performance of the 33 population pharmacokinetic models [3,4,5,15,17,19,26,27,28,29,30,31,32,33,34,35,36,37,38,39,40,41,42,43,44,45,46,47,48,49,50,51,52], ranked by the following predictive performance metrics: (**a**) mean error (ME); (**b**) relative mean error (Rel ME); (**c**) relative medium error (Rel MdE); (**d**) root mean squared error (RMSE); and (**e**) proportion within 30% of observed vancomycin concentrations (p30%). Models in the medium teal color met the a priori threshold for the metric, while the models in light red failed to meet the threshold.

A total of 33 distinct popPK models [3,4,5,15,17,19,26,27,28,29,30,31,32,33,34,35,36,37,38,39,40,41,42,43,44,45,46,47,48,49,50,51,52], including our previously published Chung 2023 [19] model, were included in the evaluation. These models encompass a range of structural assumptions (one- vs. two-compartment), and covariates (weight, age, SCr, etc.) (see Supplementary Table S2 for a summary of equations used in the derived popPK models). Overall, the majority of the popPK models failed to meet most of the predefined performance criteria (Figure 1). Older models such as the Seay 1994 [52], Rodvold 1995 [51] and Capparelli 2001 [47] models showed the highest bias. Further descriptions of the popPK models’ performances are found in the Supplementary Materials.

Table 5 summarizes the error metrics for the six best-performing models that met at least three of the five performance criteria (detailed results for all 33 models are provided in Supplementary Table S3). All top six models had relatively low RMSE (8–9.4) and rel MdE within ±13%, indicating that they generally predict typical vancomycin concentrations reasonably well. The Chung 2023 [19] model demonstrated the highest overall predictive accuracy. It had small bias (ME −0.46 mg/L, Rel ME 12.2% and Rel MdE 4.2%) and the least imprecision (RMSE 8). It had among the highest proportion of predictions from this model, falling within 30% of the true observed concentration (p30 = 49.3%). Five models met three or four of the criteria and were considered to be the next-best: Oudin 2011 [42] met four criteria, while Jung 2021 [27], Germovsek 2019 [32], Marqués-Miñana 2010 [44], and De Hoog 2000 [48] met three criteria. Common structural patterns among the top six models were one compartment and all incorporated body weight as a covariate for both CL and V. The majority also included PMA as covariates on CL (4/5 models); while two of them (Chung 2023 [19] and Oudin 2011 [42]) included SCr to account for kidney function differences.

**Figure 2:**
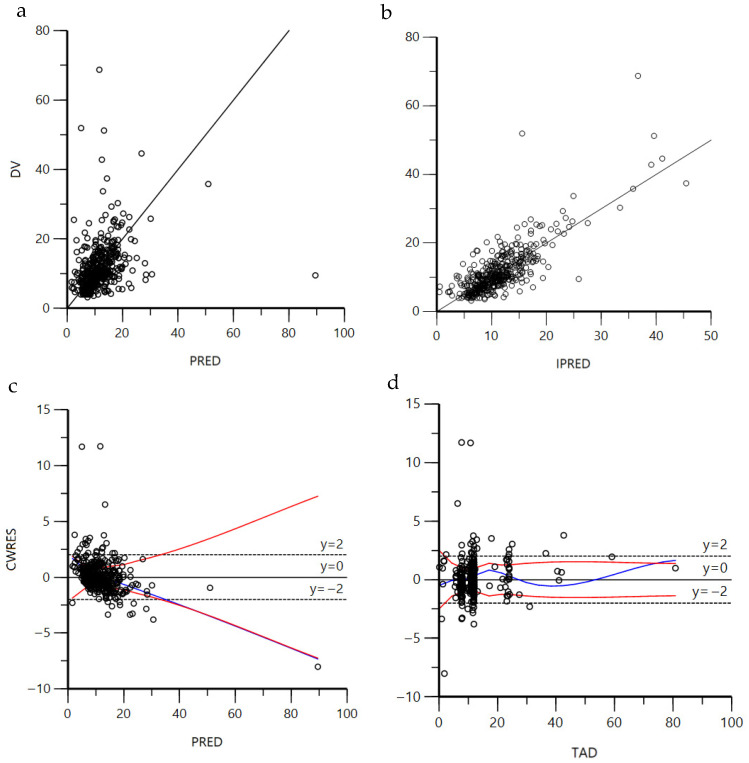
Visual predictive diagnostics for Chung 2023 [19] population pharmacokinetic model when including only the first 7 days of vancomycin therapy for each neonate, including (**a**) observed concentrations (DV) versus population predictions (PRED) with a line of identity to assess concordance; (**b**) observed concentrations (DV) versus individual predictions (IPRED); (**c**) conditional weighted residuals (CWRES) versus population predictions; and (**d**) conditional weighted residuals (CWRES) vs. time after dose (TAD in hours), including a horizontal reference line at zero. The blue lines are the smoothed locally weighted scatter plot trend lines of all values, and red lines are the absolute regression trend lines.

**Figure 3:**
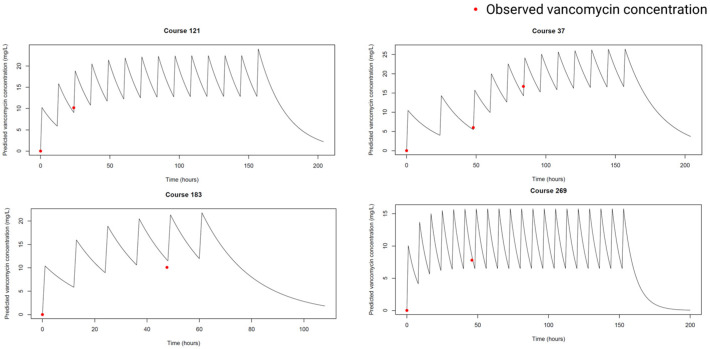
Selected predicted concentration–time profiles from the cohort, based on the Chung 2023 model [19].

Comparison of model fit diagnostics for the top six population pharmacokinetic models demonstrated both similarities and some differences in predictive performance and residual behavior (Supplementary Figure S1). The DV-PRED and residual plots appear to be similar between all models, but the Chung 2023 [19] model showed a slightly tighter clustering of observations around the identity line. Residuals were more evenly distributed around zero across both the time and predicted concentration ranges, suggesting lower time- or prediction-dependent bias. These diagnostic patterns indicate a generally slightly better fit and improved predictive consistency for the Chung 2023 [19] model, relative to the other models. Figure 2 shows the diagnostic plots when the cohort data are filtered to only up to 7 days of course to minimize the impact of time-varying maturation. The VPC plot demonstrates that most of the observations fell within the simulated percentiles (Supplementary Figure S2). Figure 3 shows the simulated concentration–time profiles based on the Chung 2023 [19] model, which demonstrated that the Chung 2023 model predicted the observed vancomycin concentrations well.

**Figure 4:**
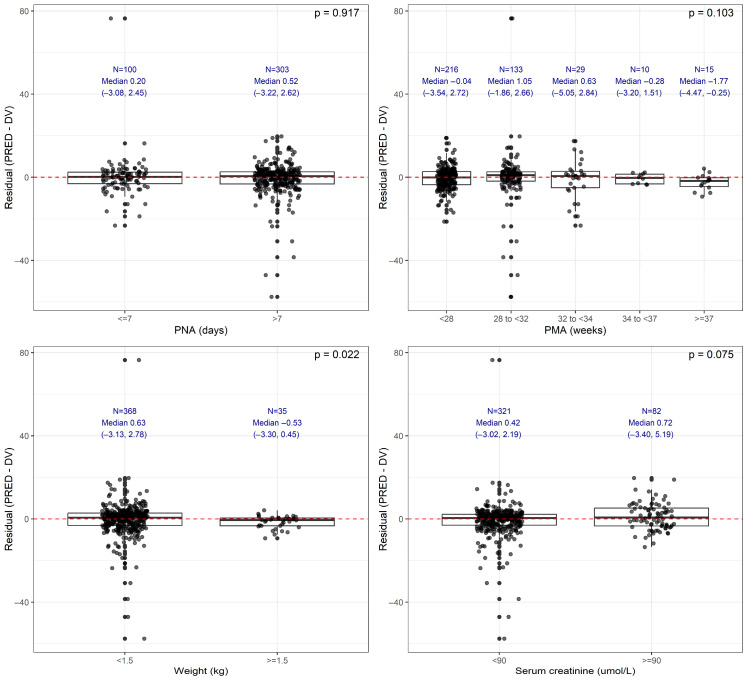
Residual error (PRED − DV) stratified by four clinically relevant neonatal covariates: postnatal age (PNA), postmenstrual age (PMA), weight (WT), and SCr. Each panel displays boxplots overlaid with a red dashed line indicating zero prediction error. Group-level summary statistics (*N*, median with interquartile range [Q1, Q3]) are shown above each category. PNA was categorized as ≤7 vs. >7 days; PMA as <28, 28 to <32, 32 to <34, 34 to <37, and ≥37 weeks; weight as <1.5 vs. ≥1.5 kg; and iSCr as <90 vs. ≥90 µmol/L. Statistical comparisons between groups were performed using Mann–Whitney U tests (for 2 groups) or Kruskal–Wallis tests (for >2 groups), and the corresponding *p*-values are displayed in the upper right corner of each panel.

Using the Chung 2023 model [19], the residual error was consistent across most clinical subgroups evaluated, including the postnatal age, the expanded five tier PMA classification, and the SCr categories, with median residuals near zero and overlapping IQR (Figure 4). In contrast, weight was the only variable for which a statistically significant difference in residual distributions was observed. Infants weighing <1.5 kg demonstrated greater variability and a modest shift in residual distribution relative to those ≥1.5 kg. Although this difference was statistically significant, the median values remained close to zero in both groups, suggesting that any weight-related bias was small in magnitude. Although some strata, particularly the PNA > 7 days, very preterm group (28 to <32 weeks), and SCr < 90 µmol/L, exhibited wider variability, no statistically significant difference was observed. Overall, these results indicate that the model performed robustly across the key neonatal subgroups, with only body weight showing a detectable impact on the residual behavior.

## 4. Discussion

This study presents an evaluation of vancomycin TDM and associated clinical outcomes in neonates, revealing substantial variability in vancomycin concentrations. Furthermore, higher vancomycin concentrations > 15 mg/L were not associated with improved effectiveness outcomes (i.e., persistent positive culture, infection recurrence, or mortality), but were significantly associated with AKI, underscoring the importance of balancing efficacy and safety in neonatal dosing strategies. Furthermore, through rigorous external validation, we identified a locally developed popPK model that significantly outperformed 32 other published models, offering a robust tool for precision dosing in this vulnerable population.

**Clinical implications of current dosing practices:** Our findings highlight an important disconnect between the existing neonatal vancomycin dosing approach and achieving the optimal therapeutic target ranges. We observed that nearly three-quarters of the initial vancomycin trough concentrations were outside the 10–15 mg/L range. These results indicate that the hospital’s empiric dosing often missed the mark, with the majority of the initial vancomycin concentrations being below the target range. Conversely, infants who were more preterm, had lower birth weights, or exhibited higher baseline SCr were significantly associated with a higher risk of supratherapeutic concentrations (>15 mg/L), which suggests an increased AKI risk without a clear added benefit in infection clearance.

**Clinical implications of current monitoring practices:** This AKI risk aligns with previous published work. In our previous work, we found improvement in bacteriological cure rates with mean vancomycin troughs above 8–10 mg/L, but did find higher AKI rates when troughs exceeded 15 mg/L [8]. Our analysis showed a higher percentage of vancomycin courses with persistent positive culture when the initial vancomycin concentration was <10 mg/L compared to above 10 mg/L, but it was not statistically significant. The absence of statistical significance in this analysis is likely due to the small number of adverse events observed in our cohort, which limits the study’s power to detect associations and adjust for possible confounders. This lower incidence may be partly explained by clinicians adjusting vancomycin doses based on the TDM results, as well as differences in the target trough ranges between the current and previous study. In our current neonatal cohort, vancomycin was dosed to achieve trough concentrations of 10–15 mg/L, whereas the previous study used a lower target range of 5–12 mg/L for neonatal sepsis. The latter found that spending longer periods with low vancomycin troughs was linked to a higher risk of adverse outcomes (adjusted OR 1.36, *p* = 0.04) [8]. Because our current analysis used a fixed target threshold, future research that examines alternative cutoffs may provide additional insight.

This study demonstrated that the commonly cited adult trough target of 15–20 mg/L for serious MRSA infections (bacteremia, meningitis, endocarditis, osteomyelitis, pneumonia, or severe skin and soft tissue infections) [7] may not be appropriate in neonates, who appear to be more vulnerable to vancomycin-induced nephrotoxicity and may achieve therapeutic success with lower vancomycin trough concentrations, such as targeting near 10 mg/L. In our study, neonates with a vancomycin trough >15 mg/L experienced a significantly higher incidence of AKI (20.8%) compared to those with troughs <10 mg/L (0.8%) or 10–15 mg/L (6.6%) (*p* < 0.001), without any statistically significant reduction in persistent positive culture or mortality. This suggests that the AKI risk associated with the adult-targeted range outweighs its efficacy benefits in our cohort. While our study was not powered to stratify outcomes by specific infection types, these findings support a more conservative trough target range for common neonatal sepsis; however, caution is advised when treating severe MRSA infections with higher MICs, where higher targets might still be clinically warranted despite the increased AKI risk. In addition, our findings highlight the complexity of dosing vancomycin in neonates, as even minor variations in maturation and renal function can lead to significant pharmacokinetic differences. By using a popPK model that incorporates these individual factors, clinicians can achieve more accurate and personalized dosing, helping them to optimize therapeutic outcomes and minimize the risk of toxicity or sub-therapeutic exposure in this vulnerable population.

**Model evaluation and comparative performance:** Our comparative analysis showed that not all published popPK models perform equally well; some, despite widespread use, exhibited substantial bias when applied to this cohort. In contrast, the Chung 2023 [19] model, which was developed locally within the same city but at a different institution, delivered superior predictive performance, even with significant differences in neonatal characteristics between the cohort used to develop the model and the cohort under evaluation. The Chung 2023 [19] model, which incorporated weight-standardized CL maturation and renal function and weight-standardized V, predicted vancomycin concentrations in the external Level III NICU with high accuracy (overall <0.5 mg/L bias). It outperformed other published models, including several contemporary ones, in forecasting vancomycin concentrations. This superior performance likely reflects both the model’s covariate design and the population similarity: our model was derived from a large dataset from a Canadian NICU, and we validated it in a cohort from the same region and practice environment. It is encouraging that a model developed in a higher-acuity NICU (Level IV) remained accurate in a lower-acuity NICU (Level III), despite significant baseline differences. While models like Oudin 2011 [42] and Germovsek 2019 [32] demonstrated good generalizability to the same Canadian cohort, the superior performance of the Chung 2023 [19] model may partly reflect the shared regional practices and neonatal demographics. Further cross-institutional validation is required to confirm its superiority in distinctly different healthcare settings. These findings demonstrated that the selected covariates (weight, PMA, and SCr) effectively capture the pharmacokinetic variability across different levels of care and clinical severities and support the generalizability of the model for broader application in varied NICU settings, not limited to the original level IV care population.

Top-performing models in our evaluation used one-compartment structures with allometric weight scaling and maturation based on PMA and/or SCr as a covariate for clearance, underscoring that these features are crucial for neonatal PK modeling. The superior performance of one-compartment models over more complex two-compartment structures likely reflects the data structure of our study, which relied exclusively on trough concentrations. Without early peak sampling, the distribution phase is poorly characterized, making two-compartment models prone to over-parameterization and instability, whereas the parsimonious one-compartment approach provided robust and reliable predictions for the elimination phase that drives clinical dosing decisions. One interesting observation was that certain models excelled in one performance aspect but not others. For example, the Oudin 2011 [42] model had exceptionally low Rel ME (only ~3%) in our data, yet its slight bias (a tendency to under-predict, with a ME of −1.1 mg/L) kept it from meeting our strict criteria for bias. Jung 2021 [27] achieved virtually zero ME, implying that it captured the average CL well, but had broader scatter (Rel ME ~22%), which was possibly due to not accounting for SCr variability. These nuances demonstrate that model evaluation should not be limited to a single metric, but should examine multiple approaches to ensure certainty regarding a model’s ability to predict with both high accuracy and precision. Models published thirty years ago performed poorly, with greater bias and imprecision. Seay 1994 [52] and Rodvold 1995 [51] had ME of 10.1 mg/L and −11.5 mg/L, respectively and p30 of only 24% and 0.3%, respectively, showing limited relevance for modern extremely preterm infants. This was likely due to outdated nonlinear mixed-effects algorithms, differences in SCr assays (Jaffe vs. isotope dilution mass spectrometry-traceable or enzymatic methods), and improved popPK analysis methods. Collectively, these discrepancies highlight that model failure in external validation is often multifactorial, stemming from significant differences in the developmental stage of the study population (extremely preterm vs. term infants), the omission of critical covariates such as renal function markers (SCr), or the use of outdated analytical methods, whereas the Chung 2023 [19] model succeeded by aligning these key elements with the target population.

Indeed, previous external evaluations by Blouin et al. also reported that no more than a few models met all their acceptance criteria, and that different models performed similarly within a narrow band of error [21]. Our work extends those findings by examining a larger set of models (33 vs. 28 in Blouin’s report) and employing slightly different criteria that emphasize absolute prediction error measured in mg/L. Notably, Blouin et al. identified two models (Capparelli 2001 [47] and Mehrotra 2012 [41]) as optimal, based on their analysis. However, within our dataset, the Capparelli 2001 [47] model performed among the least effectively, while the Mehrotra 2012 [41] model demonstrated an average performance. These discrepancies may stem from differences in the external populations or evaluation methods; our cohort contained a larger proportion of extremely preterm neonates with low renal clearance. Capparelli’s 2001 [47] model was developed in relatively more mature neonates, with mean GA of 33.5 weeks and PNA of 70 days up to 2 years of age, and indeed in Blouin’s external set with more mature neonates (mean PMA ~35 weeks), they performed adequately, but in our external cohort (mean PMA ~29 weeks), it dramatically over-predicted the vancomycin concentrations, leading to high bias. Such findings emphasize that even published neonatal popPK models are not universally transferable and should be validated before clinical use in a new center. Encouragingly, some models did generalize well beyond their original setting. The Oudin 2011 [42] and Germovsek 2019 [32] models, though developed in Europe, generalized well to the neonates included in our study. Both models evaluated vancomycin given as continuous infusions and included infants with a mean PMA of 33 weeks and a median PMA of 29 weeks, respectively. These findings reinforced that models with appropriate allometric scaling and renal function adjustments can be transported across settings and suggests that the model structure, covariates included, and the characteristics of the study population used to develop the model may matter more than geographic population differences.

**Strengths and limitations:** Our study has several strengths: the largest neonatal cohort to date, rigorous external validation of numerous models, and a comprehensive outcome assessment. To our knowledge, this is the most extensive head-to-head comparison of vancomycin PK models in neonates, and it used stringent criteria, reflecting the clinically relevant error thresholds. We have provided quantitative evidence that one model yields superior predictive performance in our setting. We also confirmed that achieving a higher vancomycin trough target range above 15 mg/L does not necessarily equate to better outcomes in neonates, reinforcing a shift toward lower vancomycin concentrations and SCr monitoring.

There are also limitations that are inherent to its retrospective, single-center design. First, the study relied exclusively on trough concentrations, since peaks were not routinely measured in our NICUs, which precluded an AUC-based exposure analysis. While troughs are commonly used as surrogates for the AUC in current practice, they may not fully capture the total drug exposure, particularly for models that predict peak concentrations differently. Second, specific administration times for every dose were not consistently recorded; we only had the start and end times of each regimen. This necessitated assumptions about dosing intervals, introducing potential inaccuracies in assigning sampling times that were relative to the last dose, which may have contributed to the bias observed in certain courses. While the urine output data were consistently available for all neonates, approximately 30% lacked a documented baseline SCr, which limited comprehensive AKI assessment, and they were unable to be used in the popPK evaluation. AKI is multifactorial. While we collected inflammatory markers and concomitant nephrotoxic medication use, these were not used as a multivariable model to evaluate outcomes due to the limited sample size. The external validation cohort came from a single Level III NICU with specific local practices; while this allowed for a focused comparison, it may not capture the full heterogeneity of neonatal units worldwide. In addition, both the model development and validation datasets were derived from Canadian NICU populations. While vancomycin is predominantly eliminated via glomerular filtration (making ethnic or genetic metabolic polymorphisms less likely to impact pharmacokinetics compared to hepatically cleared drugs), differences in healthcare system practices, such as fluid management protocols, and local assay characteristics may influence the drug exposure and model performance in other settings. Nonetheless, we included models from Asia, Europe, and North America to test their applicability. The retrospective nature of the study also limits our ability to control for unmeasured confounding variables, such as subtle variations in hemodynamic support which could influence vancomycin clearance. Additionally, our performance criteria, though grounded in the prior literature, are somewhat arbitrary. Different thresholds might label a different set of “best” models. We chose strict cutoffs to identify truly minimal-bias models, as even small average errors can matter in dosing fragile neonates. Furthermore, the sampling site information is unavailable. Therefore, sample contamination cannot be ruled out, particularly if samples were collected from the same intravenous line. Finally, while we demonstrated improved pharmacological surrogates with model-based dosing, our study was not powered to conclusively show improved clinical outcomes; a prospective trial would be ideal to confirm a specific target range, and that precision dosing yields superior clinical outcomes (e.g., faster cure, less nephrotoxicity).

## 5. Conclusions

Optimizing neonatal vancomycin therapy remains a significant clinical challenge. Our findings suggest that targeting vancomycin trough concentrations above 15 mg/L was not associated with improved efficacy, but was associated with increased AKI incidence. A more conservative trough target near 10 mg/L may offer a safer balance for common neonatal sepsis. In rigorous external validation including extremely preterm infants, the Chung 2023 [19] model consistently outperformed over thirty published models, offering a promising approach for individualizing initial vancomycin dosing. However, prospective multicenter trials are essential to confirm the clinical utility of this precision dosing strategy and to verify that it leads to improved neonatal outcomes, such as reduced nephrotoxicity and effective infection clearance, before widespread implementation.

## Figures and Tables

**Table 1:** Neonate characteristics of the current study cohort and previous cohort used for model development.

	Current Study Cohort (*N* = 366)	Previous Cohort [19] Used for popPK Model Development (*N* = 648)	Overall (*N* = 1014)	*p*-Value
**Demographics**				
Male, *n* (%)	202 (55.2)	381 (58.8)	583 (57.5)	0.54
Weight (kg), mean (SD)	1.06 (0.60)	1.88 (0.99)	1.58 (0.95)	**<0.001**
Birth weight (kg), mean (SD)	0.87 (0.54)	1.49 (1.04)	1.27 (0.94)	**<0.001**
Gestational age (weeks), mean (SD)	26.4 (3.22)	29.9 (5.52)	28.6 (5.11)	**<0.001**
Postmenstrual age (weeks), mean (SD)	28.9 (3.81)	34.3 (5.03)	32.4 (5.32)	**<0.001**
Postnatal age (days), median (IQR)	12.0 (8.00, 21.0)	21.3 (9.33, 44.3)	17.0 (8.10, 36.0)	**<0.001**
**Clinical characteristics**				
Serum creatinine (μmol/L), median (IQR)	56.0 (39.0, 79.5)	29.0 (21.0, 43.0)	36.0 (24.0, 56.0)	**<0.001**
Last 24 hr urine output (mL/kg/hr), mean (SD)	3.89 (1.56)	3.83 (1.68)	3.85 (1.64)	0.95
Intra-abdominal infection, *n* (%)	96 (26.2)	332 (51.2)	428 (42.2)	**<0.001**
PDA, *n* (%)	159 (43.4)	160 (24.7)	319 (31.5)	**<0.001**
Congenital heart disease (other than PDA), *n* (%)	5 (1.4)	83 (12.8)	88 (8.7)	**<0.001**
**Concurrent use of nephrotoxic drugs**				
Aminoglycoside (gentamicin or Tobramycin), *n* (%)	231 (63.1)	338 (52.2)	569 (56.1)	**0.003**
Furosemide, *n* (%)	68 (18.6)	198 (30.6)	266 (26.2)	**<0.001**
Amphotericin B, *n* (%)	21 (5.7)	20 (3.1)	41 (4.0)	0.131
Acyclovir, *n* (%)	0 (0)	28 (4.3)	28 (2.8)	**<0.001**
Hydrochlorothiazide-spironolactone, *n* (%)	0 (0)	10 (1.5)	10 (1.0)	0.035

IQR = interquartile range; PDA = patent ductus arteriosus; and SD = standard deviation. Tests: For numeric variables, the Wilcoxon rank-sum test was used when the grouping factor had exactly two levels and a Kruskal–Wallis test was performed when the grouping factor contained more than two levels. For categorical variables, Fisher’s exact test was applied to the contingency table of outcomes versus groups.

**Table 2:** Baseline characteristics stratified by initial vancomycin trough concentration groups.

Initial Vancomycin Concentrations	<10 mg/L	10–15 mg/L	>15 mg/L	*p*-Value
*N* = 191 Neonates (≥5 Days of Vancomycin Therapy)	*n* = 114	*n* = 55	*n* = 22	
Female sex, *n* (%)	52 (45.6)	26 (47.3)	11 (50.0)	0.69
Birth weight (kg), median (IQR)	0.76 (0.61, 0.97)	0.68 (0.60, 0.88)	0.71 (0.59, 0.81)	0.23
Gestational age (weeks), median (IQR)	26.3 (24.7, 27.9)	25.0 (23.8, 27.1)	24.8 (24.0, 25.8)	0.008
SNAPPE II at birth, mean (SD)	41 (29, 58)	51 (26, 53)	51 (36, 65)	<0.001

IQR = interquartile range; SD = standard deviation; and SNAPPE II = score for neonatal acute physiology with perinatal extension-II. Tests: Kruskal–Wallis rank sum test for numerical variables and Fisher’s exact test for categorical variables.

**Table 3:** Clinical characteristics at vancomycin course initiation.

Initial Vancomycin Concentrations	<10 mg/L	10–15 mg/L	>15 mg/L	*p*-Value
*N* = 216 Vancomycin Courses (≥5 Days)	*n* = 131	*n* = 61	*n* = 24	
PNA, median (IQR), days	14 (10, 24)	9 (7, 15)	14 (8, 17)	0.004
PMA, median (IQR), weeks	28.7 (26.9, 31.1)	26.7 (25.6, 29.9)	26.7 (25.5, 28.2)	<0.001
Weight at first dose, median (IQR), kg	0.92 (0.76, 1.21)	0.77 (0.65, 1.14)	0.78 (0.67, 0.92)	0.009
Baseline SCr, median (IQR), µmol/L	42 (29, 55)	70 (56, 88)	76 (59, 100)	<0.001
Baseline urine output, mean (SD), mL/kg/hr	4.1 (1.4)	3.8 (1.9)	3.6 (1.5)	0.02
Central venous line in situ, *n* (%)	73 (55.7)	35 (57.4)	12 (50.0)	0.84
Concurrent aminoglycoside use, *n* (%)	86 (65.5)	32 (52.4)	18 (75.0)	0.09
Concurrent furosemide use, *n* (%)	18 (13.7)	20 (32.8)	11 (45.8)	<0.001

PMA = postmenstrual age; PNA = postnatal age, and SCr = serum creatinine. Tests: Kruskal–Wallis rank sum test for numerical variables and Fisher’s exact test for categorical variables.

**Table 4:** Clinical outcomes stratified by the initial trough vancomycin concentration.

Initial Vancomycin Concentrations	<10 mg/L	10–15 mg/L	>15 mg/L	*p*-Value
*N* = 216 Vancomycin Courses	*n* = 131	*n* = 61	*n* = 24	
Persistent positive culture, *n* (%)	6 (4.6)	2 (3.3)	0	0.88
Recurrent infection, *n* (%)	0	1 (1.6)	0	NA
All-cause mortality, *n* (%)	5 (3.8)	6 (9.8)	2 (8.3)	0.95
Nephrotoxicity *, *n* (%)	1 (0.8)	4 (6.6)	5 (20.8)	<0.001

NA = not available. * Based on neonatal RIFLE (risk, injury, failure, loss of kidney function, and end-stage kidney disease) criteria and modified KDIGO (modified Kidney Disease Improving Global Outcomes) classification. Tests: Kruskal–Wallis rank sum test for numerical variables and Fisher’s exact test for categorical variables.

**Table 5:** Predictive performance of top six vancomycin population PK models (external validation cohort, *n* = 366 neonates, 661 concentrations).

Model	ME (mg/L) (95% CI)	Rel ME (%) (95% CI)	Rel MdE (%) (95% CI)	RMSE	p30% (%)	Number of Criteria Met
Chung 2023 [19]	−0.46 (−1.22, 0.35)	12.2 (6.4, 19.2)	4.2 (−3.1, 9.1)	8.00 (5.90, 10.30)	49.3 (44.4, 53.9)	5
Oudin 2011 [42]	−1.10 (−1.85, −0.26)	2.7 (−3.4, 10.2)	−11.7 (−17.0, −6.8)	8.37 (6.48, 10.22)	50.0 (45.1, 55.1)	4
Jung 2021 [27]	0.02 (−0.69, 0.67)	22.1 (15.8, 29.0)	7.8 (2.1, 13.6)	8.41 (6.87, 10.23)	38.3 (34.4, 42.3)	3
Germovsek 2019 [32]	−0.77 (−1.44, −0.08)	12.2 (5.7, 19.0)	0.2 (−4.9, 6.7)	8.38 (6.84, 10.23)	43.3 (39.2, 47.4)	3
Marqués-Miñana 2010 [44]	0.31 (−0.35, 1.04)	25.7 (19.0, 33.0)	12.9 (4.4, 20.0)	8.78 (7.03, 10.84)	35.5 (31.6, 39.5)	3
DeHoog 2000 [48]	−1.25 (−2.01, −0.54)	9.4 (2.1, 16.8)	−9.8 (−16.6, −3.7)	9.36 (7.77, 11.16)	34.3 (30.7, 38.0)	3

CI, confidence interval; p30, percentage within 30% of observed vancomycin concentration; ME, mean error; MdE, median error; Rel, relative; and RMSE, root mean square error.

## Data Availability

The data presented in this article are not readily available because of privacy and ethical reasons. Requests to access the data should be directed to the corresponding author.

## Supplementary Materials

The following supporting information can be downloaded at: https://www.mdpi.com/article/10.3390/children13050649/s1. Further descriptions of the popPK analyses, as well as supplementary tables and figures, are available in the Supplementary Materials, which include the following:

- Further description for 3.4. Predictive performance of population pharmacokinetic models
- Supplementary Table S1. Neonatal RIFLE criteria and modified KDIGO classification.
- Supplementary Table S2. Equations of Population Pharmacokinetic Models of Vancomycin in Neonates.
- Supplementary Table S3. Summary of Predictive Performance Metrics of the 33 population pharmacokinetic models.
- Supplementary Figure S1. Visual predictive diagnostics for the top population pharmacokinetic models. Each row corresponds to one model and displays: observed concentrations (DV) versus population predictions (PRED) with a line of identity to assess concordance; residuals versus time (hours), including a horizontal reference line at zero to evaluate time-related trends; and residuals versus PRED, again with a zero-residual reference line to assess potential prediction-related bias. Together, these panels provide a qualitative comparison of goodness-of-fit between the two models.
- Supplementary Figure S2. Visual Predictive Check Plot based on Chung 2023 [19] model.

## Abbreviations

The following abbreviations are used in this manuscript:

AKI	Acute kidney injury
CI	Confidence interval
CL	Clearance
CoNS	Coagulase-negative Staphylococci
*C*_pred_ or PRED	Predicted concentrations
*C*_obs_ or DV	Observed concentrations
GA	Gestational age
IQR	Interquartile range
ME	Mean prediction error
MIPD	Model-informed precision dosing
MRSA	methicillin-resistant *Staphylococcus aureus*
*n*	Number of neonates in a subgroup
*N*	Total number of neonates in study cohort
NICU	Neonatal intensive care unit
OR	Odds ratio
PE	Prediction error or residual
PK	Pharmacokinetic
popPK	Population pharmacokinetic
PMA	Postmenstrual age
PNA	Postnatal age
Rel MdE	Relative median prediction error
Rel ME	Relative mean prediction error
RMSE	Root mean squared error
SCr	Serum Creatinine
SNAPPE-II	Score for Neonatal Acute Physiology II
SD	Standard deviation
TDM	Therapeutic drug monitoring
V	Volume of distribution
WT	Weight
