# Supplementary material for: Vancomycin Therapeutic Drug Monitoring, Clinical Outcomes and Population Pharmacokinetic Model Evaluation in Neonates
Source: Children (Basel). 2026 May 6;13(5):649. doi: 10.3390/children13050649 (PMC13204513; doi:10.3390/children13050649)
Supplement: Supplementary file 1 [file children-13-00649-s001.zip › children-4204145-Supplementary Materials_Apr30_2026.pdf]

Vancomycin Therapeutic Drug Monitoring, Clinical Outcomes and Population Pharmacokinetic Model Evaluation in Neonates

Supplementary Materials

Erin Chung [1,2,3], Najla Tabbara [4], Winnie Seto [1,2,3,5] and Vibhuti Shah [5,6,*]

[1] The Hospital for Sick Children, 555 University Avenue, Toronto, ON M5G 1X8, Canada; erin.chung@utoronto.ca (E.C.); winnie.seto@sickkids.ca (W.S.)
[2] Child Health Evaluative Sciences, SickKids Research Institute, 686 Bay Street, Toronto, ON M5G 0A4, Canada
[3] Leslie Dan Faculty of Pharmacy, University of Toronto, 144 College Street, Toronto, ON M5S 3M2, Canada
[4] Department of Pharmacy, Mount Sinai Hospital, 600 University Avenue, Toronto, ON M5G 1X5, Canada; najla.tabbara@sinaihealth.ca
[5] Institute of Health Policy, Management and Evaluation, University of Toronto, 155 College Street, Toronto, ON M5T 3M6, Canada
[6] Department of Paediatrics, Mount Sinai Hospital, 600 University Avenue, Rm 19-231N, Toronto, ON M5G 1X5, Canada

* Correspondence: vibhuti.shah@sinaihealth.ca; Tel.: 416-586-4816; Fax: 416-586-8745

Further description for *3.4. Predictive performance of population pharmacokinetic models*

Based on the mean prediction error, 4 (12%) models (Chung 2023 [19], Alsultan 2023 [17], June 2021 [27], and Marqués-Miñana 2010 [44]) met the threshold ±0.5 mg/L. While 6 (18%) models (Chung 2023 [19], Mulubwa 2020 [30], Germovsek 2019 [32], Oudin 2011 [42], DeHoog 2000 [48], Grimsley ad Thomson 1999 [49]) met the threshold for rel ME ±15%. A total of 8 (24%) models (Chung 2023 [19], Jung 2021 [27], Cristea 2019 [31], Germovsek 2019 [31], De Cock 2014 [39], Oudin 2011 [42], Marqués-Miñana 2010 [44], DeHoog 2000 [48]) met the threshold for relative median prediction error ±15%. While 18 (54%) models met the root mean squared error <10 (Chung 2023 [19], Alsultan 2023 [17], Jarugula 2022 [5], Jung 2021 [27], Lee 2021 [4], Sasano 2021 [28], Mulubwa 2020 [30], Cristea 2019 [30], Germovesk 2019 [32], Chen 2018 [32], Bhongsatiern 2015 [37], Frymoyer 2014 [3], Mehrotra 2012 [41], Oudin 2011 [41], Marqués-Miñana 2010 [44], Allegarert 2007 [45], DeHoog 2000 [48], Grimsley and Thomson 1999 [49]). Only 3 (9%) popPK models had >45% of predictions within 30% of the observed vancomycin concentrations (Chung 2023 [19], Jarugula 2022 [5], and Oudin 2011 [42]).

Seay 1994 [52] and Capparelli 2001 [47] systematically overpredicted vancomycin concentrations in the study cohort (positive bias: ME 10.1 mg/L [95% CI: 9 to 11.4] and 16.8 mg/L [95% CI: 14.3 to 19.6], respectively). While Rodvold 1995 underpredicted significantly (negative bias: ME -11.5 mg/L [95% CI: -12.3 to -10.9]).

Oudin 2011 [42] met 4 performance criteria, while Jung 2021 [27], Germovsek 2019 [32], Marqués-Miñana 2010 [44], and De Hoog 2000 [48] met 3 criteria. The Oudin 2011 [42] model was developed in 68 preterm infants with PMA of 23-41 weeks, included weight and SCr on CL and weight on V, predicted the cohort data with similar precision as Chung 2023 [19] model (RMSE 8.37) and 50% of predictions within 30% accuracy. However, Oudin 2011 [42] model showed a slight consistent bias (ME -1.10 mg/L [95% CI: -1.85, -0.26]), tending to underpredict concentrations), causing it to miss the ME ±0.5 mg/L criterion. Jung 2021 [27] had essentially zero mean bias (ME 0.02 mg/L) but larger Rel ME (22.1%), and lower proportion (38.3%)

within 30% accuracy, meeting three of five criteria. The Germovsek 2019 [32] model had very low Rel MdE (0.24%) but had modest negative bias (ME -0.77 mg/L [95% CI: -1.44, -0.08]) and slightly lower proportion (43.3%) within 30% accuracy. While the Marqués-Miñana 2010 [44] model and DeHoog 2000 [48] had the lowest p30% among the top 6 (35.5% and 34.3%, respectively).

Supplementary Table S1. Neonatal RIFLE criteria and modified KDIGO classification.

Stage	Serum creatinine	Urine output
0	No change or<br>↑ < 27 μmol/L	≥ 1.5 mL/kg/h in 24 h
1 (Risk)	↑ 1.5 to <2 times previous level or<br>↑ ≥ 27 μmol/L	1 to <1.5 mL/kg/h in 24h
2 (Injury)	↑ 2 to < 3 times previous level	0.7 to < 1 mL/kg/h in 24h
3 (Failure)	↑ ≥ 3 times previous level or<br>Rise ≥ 220 μmol/L	< 0.7 mL/kg/h in 24h or anuric for 12 hours
Loss	Persistent failure (stage 3) > 4 weeks	
End-stage	Persistent failure (stage 3) > 3 months	

Supplementary Table S2. Equations of Population Pharmacokinetic Models of Vancomycin in Neonates.

Study	Clearance Equation	Volume of distribution Equation
Chung 2023 [19]	$CL = 16.1 \times \left(\frac{WT}{70}\right)^{1} \times \left(\frac{PMA^{1.07}}{PMA^{1.07} + 42^{1.07}}\right) \times \left(\frac{SCr^{\neq}}{34}\right)^{-0.65}$	$V = 62.2 \times \left(\frac{WT}{70}\right)$
Alsultan 2023 [17]	$Cl = 0.09 \times \left(\frac{WT}{0.93}\right)^{0.75} \times \left(\frac{0.6}{SCr^{\ddagger}}\right)^{0.48} \times \frac{PMA^{4.42}}{PMA^{4.42} + 26.3^{4.42}}$	$V = 0.81 \times \left(\frac{WT}{0.93}\right)$
Hui 2022 [26]	$CL = 0.14 \times \left(\frac{WT}{1.7}\right)^{0.75} \times \left(\frac{(PMA\ in\ days)^{1.02}}{(PMA\ in\ days)^{1.02} + 197^{1.02}}\right) \times \left(\frac{40}{SCr^{\neq}}\right)^{0.541}$<br>$Q = 0.147 \times \left(\frac{WT}{1.7}\right)^{0.75}$	$V_c = 0.769 \times \left(\frac{WT}{1.7}\right)$<br>$V_p = 0.285 \times \left(\frac{WT}{1.7}\right)$
Jarugula 2022 [5]	$CL = 0.237 \times \left(\frac{WT}{3.5}\right)^{0.75} \times \left(\frac{0.45}{SCr^{\ddagger}}\right)^{0.87} \times \left(\frac{PMA}{42}\right)^{0.81}$	$V = 2.98 \times \left(\frac{WT}{3.5}\right)$
Jung 2021 [27]	$CL = 3.42 \times \left(\frac{WT}{70}\right)^{0.75} \times \left(\frac{PMA^{3.68}}{PMA^{3.68} + 31.2^{3.68}}\right)$	$V = 50.9 \times \left(\frac{WT}{70}\right)$
Lee 2021 [4]	$CL = 2.09 \times \left(\frac{WT}{70}\right)^{0.75} \times \left(\frac{PMA}{31.7}\right)^{0.795} \times \left(\frac{CrCL}{50.3}\right)^{0.741}$<br>*CrCL calculated based on Schwartz equation (1984)*<br>$eGFR\ (mL/min/1.73m^2) = \frac{0.45\ \times Height\ (cm)}{SCr\ (\mu mol/L) \times 0.0113}$<br>Schwartz, G. J., Feld, L. G. & Langford, D. J. A simple estimate of glomerular filtration rate in full-term infants during the first year of life. J. Pediatr. 104, 849–854 (1984).	$V = 45.6 \times \left(\frac{WT}{70}\right)$
Sasano 2021 [28]	$CL = 0.056 \times \left(\frac{WT}{0.887}\right)^{0.75} \times \left(\frac{0.35}{SCr^{\ddagger}}\right)^{0.539}$	$V = 0.827 \times \left(\frac{WT}{0.887}\right)$
Dao 2020 [29]	$CL = 0.273 \times WT^{0.438} \times \left(\frac{54}{SCr^{\neq}}\right)^{0.473} \times \left(\frac{PMA^{3.54}}{PMA^{3.54} + 46.4^{3.54}}\right)$	$V = 0.628 \times WT$
Mulubwa 2020 [30]	$CL = 0.102 \times \left(\frac{WT}{1.48}\right)^{0.75}$	$V = 0.884 \times \left(\frac{WT}{1.48}\right)$
Cristea 2019 [31]	$CL = 0.053 \times \left(\frac{BW}{1.76}\right)^{1.34} \times \left(1 + \left(0.213 \times \left(\frac{PNA}{2}\right)\right)\right) \times F_{ibu} \times F_{indo}$<br>$F_{ibu}$ =0.838 *(if concurrent use of ibuprofen)*<br>F_{indo} =0.447 *(if concurrent use of indomethacin)*<br>$Q = 0.904 \times CL$	$V_C = 0.913 \times \left(\frac{WT}{1.75}\right)^{0.919}$<br>$V_C = V_P$
Germovsek 2019 [32]	$CL = 5.7 \times \left(\frac{WT}{70}\right)^{0.632} \times \left(\frac{PMA^{3.4}}{PMA^{3.4} + 47.7^{3.4}}\right)$	$V = 39.3 \times \left(\frac{WT}{70}\right)$
Reilly 2019 [15]	$CL = 0.0558 \times WT \times \left(\frac{PMA}{30}\right)^{1.26} \times \left(\frac{PNA}{18}\right)^{0.104} \times \left(\frac{urine\ output}{3.8}\right)^{0.505}$	$V = 0.491 \times WT$
Chen 2018 [33]	$CL = 4.87 \times \left(\frac{WT}{70}\right)^{0.75} \times \left(\frac{PMA^{4.61}}{PMA^{4.61} + 34.5^{4.61}}\right) \times \left(\frac{SCr^{\ddagger}}{0.28}\right)^{-0.221}$	$V = 40.7 \times \left(\frac{WT}{70}\right)$
Li 2018 [34]	$CL = 0.309 \times \left(\frac{WT}{2.9}\right)^{1.55} \times \left(\frac{23.3}{SCr^{\neq}}\right)^{0.337}$	$V = 2.63 \times \left(\frac{WT}{2.9}\right)^{1.05}$

Study	Clearance Equation	Volume of distribution Equation
Tseng 2018 [35]	$CL = 0.0519 \times (WT)^{0.75} \times \left(\frac{PMA}{30.1}\right)^{2.4} \times \left(\frac{43}{SCr^{\neq}}\right)^{0.246}$	$V = 0.498 \times WT$
Li 2017 [36]	$CL = 4.6 \times \left(\frac{WT}{70}\right)^{0.75} \times \left[\frac{1}{1+\left(\frac{37.6}{PMA}\right)^{5.46}}\right] \times 1.2^{\left(\frac{30}{SCr^{\neq}}\right)}$	$V = 61.1 \times \frac{WT}{70}$
Bhongsatiern 2015 [37]	$CL = 0.095 \times \left(\frac{WT}{1.5}\right)^{0.585} \times \left(\frac{eGFR}{36}\right)^{0.72} \times \frac{PMA}{33}$<br>Used modified Schwartz formula (2009)	$V = 0.905 \times \left(\frac{WT}{1.5}\right)$
Caceres Guido 2015 [38]	$k_e = 0.203 \times SCr^{\text{ⱡ}^{-0.*697}}$	$V = 0.432$
De Cock 2014 [39]	$CL = 0.053 \times \left(\frac{BW}{1.14}\right)^{1.34} \times (1 + 0.213 \times \frac{PNA}{14}) \times 0.838(ibuprofen = 1)$<br>$Q = 0.904 \times CL$	$V_c = 0.913 \times \left(\frac{BW}{1.14}\right)^{0.919}$<br>$V_c = V_p$
Frymoyer 2014 [3]	$CL = 0.345 \times \left(\frac{WT}{2.9}\right)^{0.75} \times \left(\frac{1}{1+\left(\frac{PMA}{34.8}\right)^{-4.53}}\right) \times \left(\frac{1}{SCr^{\text{ⱡ}}}\right)^{0.221}$	$V = 1.75 \times \left(\frac{WT}{2.9}\right)$
Zhao 2013 [40]	$CL = 0.0571 \times \left(\frac{WT}{1.416}\right)^{0.513} \times \left(\frac{BW}{1.01}\right)^{0.599} \times \left(1 + 0.282 \times \frac{PNA}{17}\right) \times \frac{1}{\left(\frac{SCr^{\neq}}{42}\right)^{0.525}}$	$V = 0.79 \times \left(\frac{WT}{1.416}\right)^{0.898}$
Mehrotra 2012 [41]	$CL = 0.18 \times \left(\frac{WT}{2.5}\right)^{0.75} \times \left(\frac{0.42}{SCr^{\text{ⱡ}}}\right)^{0.7} \times \left(\frac{PMA}{37}\right)^{1.4}$	$V = 1.7 \times \frac{WT}{2.5}$
Oudin 2011 [42]	$CL = 82.1 \times \frac{\left(\frac{WT}{70}\right)^{0.75}}{SCr^{\neq}}$	$V = 60.5 \times \left(\frac{WT}{70}\right)$
Lo 2010 [43]	$CL = 1 \times \left(\frac{WT}{70}\right)^{0.75} \times \left(\frac{PMA}{30}\right)^{3.16} \times [0.83(SGA) + 1.03(1 - SGA)]$<br>*SGA = 1 for SGA, 0 for appropriate for GA*	$V = 36.6 \times \left(\frac{WT}{70}\right)$<br>*V = L*<br>*WT = kg*
Marqués-Miñana 2010 [44]	$CL = 0.00192 \times PMA \times (1 + 0.65 \times Amoxicillin - clavulanic\ acid) \times WT$	$V = 0.572 \times (1 - 0.344 \times spironolactone) \times WT$
Allegaert 2007 [45]	$CL = 1.58 \times \left(\frac{WT}{70}\right)^{0.75} \times e^{[0.0456 \times (PMA-30)]} \times RF \times FNCOX \times FCL\ amikacin \times FSGA$<br>$RF = \frac{CrCl}{6\ L/h} = \frac{\frac{Creatinine\ production\ rate}{SCr^{\neq}}}{6\ L/h} = \frac{\frac{(516 \times e^{\left(0.00766 * \left(\frac{PMA-30}{52} - 40\right)\right)}}{SCr^{\neq}}}{6\ L/h}$<br>*FNCOX = 0.795 if given nonselective COX inhibitor (scaling factor)*<br>*FCL amikacin = 0.567 for scaling amikacin clearance relative to that of vancomycin*<br>*FSGA = 0.838 for scaling small for gestational age*	$V = 39.3 \times \left(\frac{WT}{70}\right)$
Kimura 2004 [46]	$CL = 0.025 \times \frac{WT}{SCr^{\text{ⱡ}}} for\ PMA < 34\ weeks$<br>$CL = 0.0323 \times \frac{WT}{SCr^{\text{ⱡ}}} for\ PMA \geq 34\ weeks$	$V = 0.66 \times WT$

Study	Clearance Equation	Volume of distribution Equation
Capparelli 2001 [47]	$CL = WT \times \left(\frac{0.028}{SCr^{\ddagger}} + 0.000127 \times PNA\ (if\ SCR < 0.7\ mg/dL) + 0.0123 \times GA28\right) + 0.006$<br>$Q = 0.0334 \times WT$<br>GA28 = 1 if GA > 28 weeks<br>GA28 = 0 if GA ≤ 28 weeks	$V_c = 0.666 \times V_{ss}$<br>$V_{ss} = 0.793 \times WT + 0.0010$<br>$V_{ss} = V_c + V_P$
DeHoog 2000 [48]	$CL = 0.057 \times WT$	$V = 0.43 \times WT$
Grimsley and Thomson 1999 [49]	$CL = 3.56 \times WT/SCr^{\neq}$	$V = 0.669 \times WT$
Burstein 1997 [50]	$CL = 0.038 \times WT$<br>$Q = 0.38 \times WT$	$V_c = 0.19 \times WT$<br>$V_{ss} = 0.48 \times WT$<br>$V_{ss} = V_c + V_P$
Rodvold 1995 [51]	*CL = 0.411 × CrCL + 0.541*<br>*CrCL calculated based on Schwartz equation (1976)*<br>$eGFR\ (mL/min/1.73m^2) = \frac{k \times Height\ (cm)}{SCr\ (\mu mol/L) \times 0.0113}$<br>where:<br>**Age** / ***k***: <1 year, preterm: 0.33; <1 year, full-term: 0.45	$V = 0.551 \times WT$
Seay 1994 [52]	*CL = 0.059 × WT ×* $(0.460)^{GA32} \times (0.643)^{dopamine}$<br>$Q = 0.0313 \times WT$<br>*GA32 = 1 if ≤ 32weeks*<br>*GA32 = 0 if > 32 weeks*<br>*Dopamine = 1 (if exposed to dopamine), 0 (if not exposed)*	$V_c = 0.44 \times WT$<br>$V_{ss} = 0.764 \times WT$<br>$V_{ss} = V_c + V_P$

Age, chronological age (years); Alb, albumin (g/dL); BMI, body mass index (kg/m^2); BSA, body surface area (m^2); BUN, body urea nitrogen (mg/dL); BW, birthweight (kg); CL, elimination clearance (L/h); CRRTUF, ultrafiltration fluid rate (mL/h); CysC, Cystatin C (mg/L); DILYSTE, dialysate flow rate (mL/h); eGFR, estimated glomerular filtration rate (mL/min/1.73 m^2); FFM, fat-free mass (kg); GA, gestational age (weeks); k_e, elimination constant (h^{-1}); PMA, postmenstrual age (weeks); PNA, postnatal age (days); Q, intercompartmental clearance (L/h); RF, renal function; SCr, serum creatinine (mg/dL‡ or μmol/L$^{\neq}$); Temp, temperature (°C); Urine output (mL/kg/h); V, volume of distribution (L); V_c, volume of distribution for central compartment (L); V_P, volume of distribution for peripheral compartment (L); V_{ss}, steady state volume of distribution (L); Volume of infusion (mL/day); WT, current weight (kg).

Supplementary Table S3. Summary of Predictive Performance Metrics of the 33 population pharmacokinetic models.

Model	ME (mg/L) (95% CI)	MdE (mg/L) (95% CI)	Rel ME (%) (95% CI)	Rel MdE (%) (95% CI)	RMSE	p30 (%)
Chung 2023	-0.46 (-1.22, 0.35)	0.42 (-0.30, 0.74)	12.2 (6.4, 19.2)	4.2 (-3.1, 9.1)	8.00 (5.90, 10.30)	49.3 (44.4, 53.9)
Alsultan 2023	0.42 (-0.43, 1.20)	1.41 (1.00, 1.80)	22.9 (15.8, 29.7)	15.8 (8.4, 20.2)	8.42 (6.16, 10.96)	42.1 (37.2, 46.9)
Hui 2022	3.84 (2.91, 4.77)	4.51 (3.88, 4.94)	57.4 (47.9, 67.3)	46.6 (36.2, 54.2)	10.42 (7.87, 13.63)	34.0 (29.1, 38.6)
Jarugula 2022	1.21 (0.34, 2.10)	1.31 (0.85, 1.81)	27.5 (20.2, 35.5)	15.9 (8.9, 21.9)	8.99 (6.94, 11.17)	46.2 (41.3, 51.3)
Jung 2021	0.02 (-0.69, 0.67)	0.74 (0.19, 1.20)	22.1 (15.8, 29.0)	7.8 (2.1, 13.6)	8.41 (6.87, 10.23)	38.3 (34.4, 42.3)
Lee 2021	2.45 (1.49, 3.36)	2.10 (1.59, 2.61)	39.8 (31.9, 48.3)	25.2 (19.0, 32.4)	9.54 (7.47, 12.30)	43.1 (38.2, 47.9)
Sasano 2021	2.52 (1.65, 3.40)	3.15 (2.43, 3.75)	45.9 (37.1, 55.5)	32.4 (21.7, 38.9)	9.58 (7.38, 12.35)	33.7 (29.1, 38.3)
Dao 2020	3.89 (2.98, 4.78)	4.22 (3.53, 4.75)	56.4 (48.5, 65.7)	43.6 (37.2, 51.3)	10.33 (8.02, 12.98)	31.3 (26.7, 35.4)
Mulubwa 2020	-3.41 (-4.14, -2.68)	-3.13 (-3.74, -2.57)	-13.9 (-20.1, -7.2)	-34.9 (-41.0, -29.9)	9.39 (8.30, 10.64)	28.5 (25.2, 32.3)
Cristea 2019	0.95 (0.16, 1.72)	0.68 (0.03, 1.19)	30.8 (22.8, 38.9)	7.1 (0.3, 14.7)	9.85 (7.73, 12.40)	37.0 (33.4, 40.8)
Germovsek 2019	-0.77 (-1.44, -0.08)	0.03 (-0.45, 0.55)	12.2 (5.7, 19.0)	0.2 (-4.9, 6.7)	8.38 (6.84, 10.23)	43.3 (39.2, 47.4)
Reilly 2019	2.61 (1.72, 3.52)	2.04 (1.51, 2.72)	45.7 (35.6, 55.8)	23.0 (16.1, 30.4)	10.99 (8.00, 14.72)	36.6 (32.4, 40.8)
Chen 2018	1.30 (0.43, 2.24)	1.96 (1.35, 2.36)	32.9 (25.1, 41.9)	18.9 (12.2, 30.3)	9.54 (7.10, 12.22)	38.2 (33.3, 43.1)
Li 2018	4.09 (3.10, 5.13)	4.68 (4.03, 5.39)	62.4 (53.0, 73.2)	50.2 (38.9, 57.8)	10.79 (8.43, 13.91)	29.6 (25.5, 33.7)
Tseng 2018	2.54 (1.60, 3.53)	3.17 (2.47, 3.78)	45.1 (36.3, 55.4)	33.0 (25.0, 40.9)	10.06 (7.31, 13.24)	35.2 (30.8, 39.8)
Li 2017	5.93 (4.82, 7.03)	6.15 (5.22, 6.67)	80.9 (69.7, 92.4)	66.9 (53.6, 77.1)	12.72 (10.48, 15.39)	24.5 (20.6, 28.6)
Bhongsatiern 2015	2.12 (1.23, 2.98)	2.04 (1.56, 2.43)	36.5 (29.3, 44.8)	21.9 (17.7, 28.1)	9.37 (7.18, 11.70)	43.3 (38.2, 48.2)
Caceres Guido 2015	-9.42 (-10.09, -8.77)	-8.33 (-8.77, -7.87)	-80.1 (-83.1, -76.3)	-88.1 (-89.4, -86.7)	11.82 (10.65, 13.12)	2.4 (1.0, 3.9)
De Cock 2014	3.26 (2.33, 4.20)	1.22 (0.65, 2.19)	59.8 (49.2, 70.6)	14.9 (7.8, 24.5)	12.70 (11.12, 14.19)	33.4 (29.4, 37.2)
Frymoyer 2014	1.38 (0.49, 2.25)	2.02 (1.39, 2.50)	32.5 (24.5, 41.2)	20.1 (13.3, 30.3)	9.31 (7.03, 11.92)	38.6 (33.7, 43.4)
Zhao 2013	3.89 (3.02, 4.81)	4.22 (3.44, 4.70)	56.4 (48.1, 66.1)	43.6 (36.8, 51.7)	10.33 (8.00, 13.42)	31.3 (26.9, 35.7)
Mehrotra 2012	1.83 (0.93, 2.79)	1.97 (1.32, 2.39)	33.5 (26.1, 42.7)	20.6 (15.5, 26.6)	9.06 (6.69, 11.61)	43.4 (38.6, 48.3)
Oudin 2011	-1.10 (-1.85, -0.26)	-1.10 (-1.38, -0.54)	2.7 (-3.4, 10.2)	-11.7 (-17.0, -6.8)	8.37 (6.48, 10.22)	50.0 (45.1, 55.1)
Lo 2010	7.21 (6.33, 8.04)	7.06 (6.53, 7.52)	99.7 (89.4, 110.4)	74.4 (66.2, 89.5)	13.10 (10.97, 15.91)	22.6 (19.5, 25.8)
Marques-Minana 2010	0.31 (-0.35, 1.04)	1.18 (0.38, 1.71)	25.7 (19.0, 33.0)	12.9 (4.4, 20.0)	8.78 (7.03, 10.84)	35.5 (31.6, 39.5)
Allegaert 2007	-3.14 (-3.94, -2.39)	-3.30 (-3.98, -2.97)	-20.9 (-27.2, -14.5)	-36.2 (-40.1, -31.3)	8.82 (7.37, 10.37)	29.6 (25.2, 34.0)
Kimura 2004	5.02 (3.94, 6.11)	3.81 (3.01, 4.40)	62.7 (52.7, 73.9)	39.0 (32.5, 47.9)	11.98 (9.78, 14.62)	33.7 (29.6, 38.3)

Model	ME (mg/L) (95% CI)	MdE (mg/L) (95% CI)	Rel ME (%) (95% CI)	Rel MdE (%) (95% CI)	RMSE	p30 (%)
Capparelli 2001	16.79 (14.30, 19.58)	9.57 (8.78, 11.22)	205.9 (174.9, 239.6)	108.2 (93.6, 129.8)	31.52 (24.89, 38.68)	23.5 (19.9, 27.9)
DeHoog 2000	-1.25 (-2.01, -0.54)	-0.92 (-1.46, -0.32)	9.4 (2.1, 16.8)	-9.8 (-16.6, -3.7)	9.36 (7.77, 11.16)	34.3 (30.7, 38.0)
Grimsley and Thomson 1999	-1.71 (-2.53, -0.90)	-2.02 (-2.42, -1.56)	-5.3 (-11.8, 1.5)	-21.2 (-25.6, -17.4)	8.60 (6.90, 10.33)	40.8 (35.9, 45.6)
Burstein 1997	6.09 (5.09, 7.03)	5.60 (4.75, 6.33)	92.7 (81.6, 104.2)	67.0 (50.8, 75.6)	13.44 (11.24, 16.26)	26.4 (22.9, 30.0)
Rodvold 1995	-11.53 (-12.25, -10.89)	-9.85 (-10.35, -9.40)	-99.6 (-100.0, -98.9)	-100.0 (-100.0, -100.0)	13.62 (12.38, 14.90)	0.3 (0.0, 0.8)
Seay 1994	10.06 (8.99, 11.40)	8.01 (7.28, 8.72)	135.8 (122.9, 151.3)	91.4 (80.3, 103.8)	17.71 (15.31, 21.25)	24.3 (21.0, 27.8)

CI, confidence interval; p30, percentage within 30% of observed vancomycin concentration; ME, mean error; MdE, median error; Rel, relative; RMSE, root mean square error.

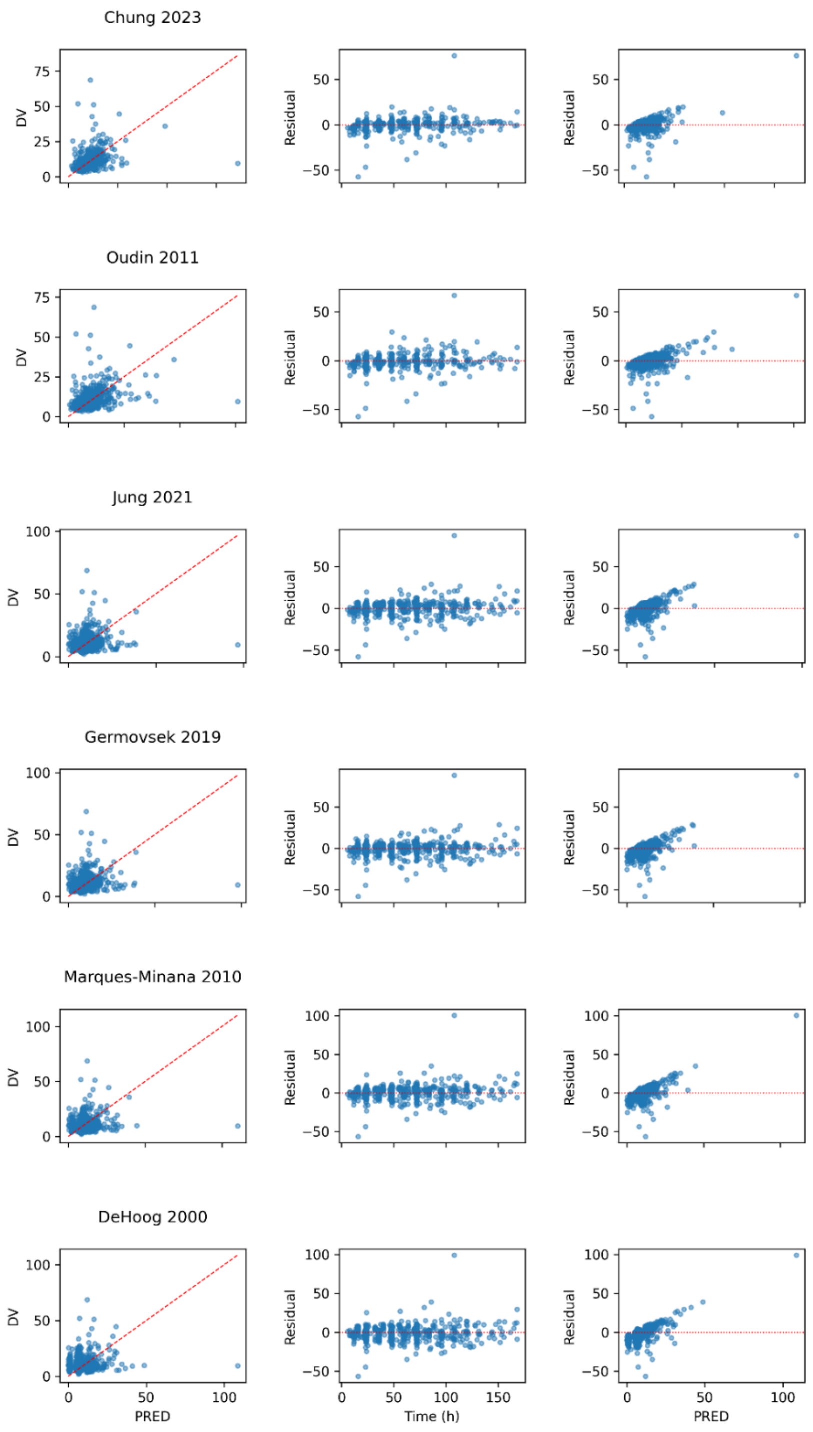

Supplementary Figure S1. Visual predictive diagnostics for the top population pharmacokinetic models. Each row corresponds to one model and displays: observed concentrations (DV) versus population predictions (PRED) with a line of identity to assess concordance; residuals versus time (hours), including a horizontal reference line at zero to evaluate time-related trends; and residuals versus PRED, again with a zero-residual reference line to assess potential prediction-related bias. Together, these panels provide a qualitative comparison of goodness-of-fit between the two models.

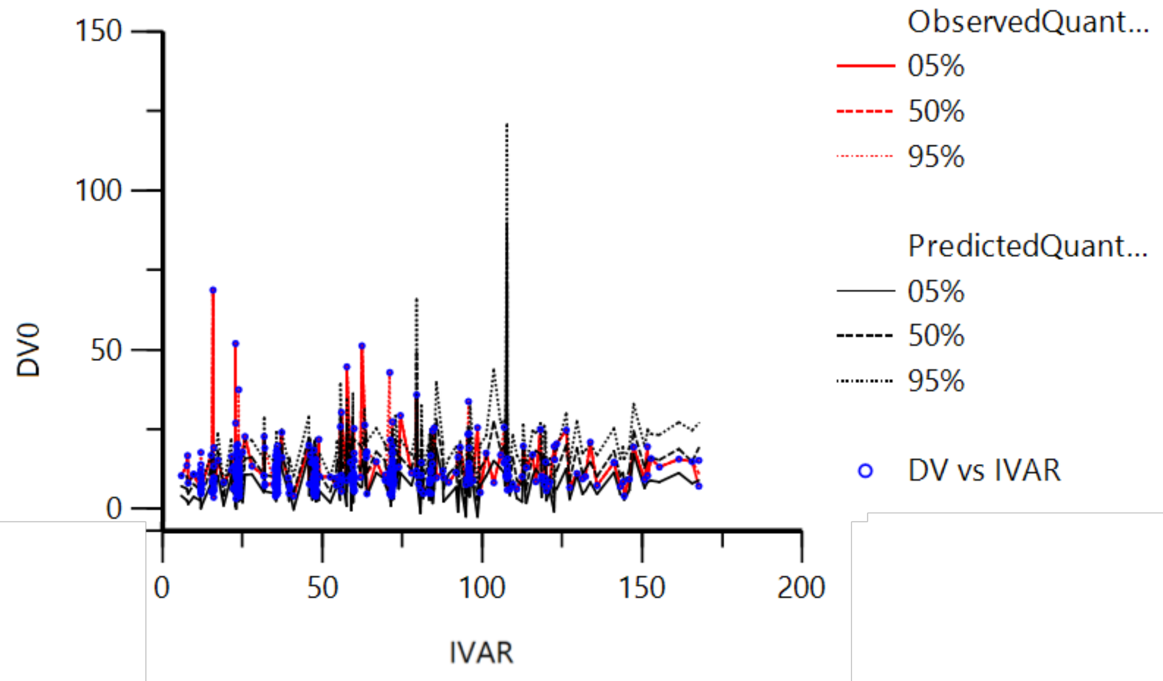

Supplementary Figure S2. Visual Predictive Check Plot based on Chung 2023 [19] model.
